# Formation of Gold Nanoclusters from Goldcarbonyl Chloride inside the Metal-Organic Framework HKUST-1

**DOI:** 10.3390/molecules28062716

**Published:** 2023-03-17

**Authors:** Zeinab Mohamed Hassan, Wei Guo, Alexander Welle, Robert Oestreich, Christoph Janiak, Engelbert Redel

**Affiliations:** 1 Karlsruhe Institute of Technology, Institute of Functional Interfaces (IFG), Hermann-von-Helmholtz-Platz 1, 76344 Eggenstein-Leopoldshafen, Germany; 2 Karlsruhe Institute of Technology, Karlsruhe Nano Micro Facility (KNMF), Hermann-von-Helmholtz-Platz 1, 76344 Eggenstein-Leopoldshafen, Germany; 3 Institut für Anorganische Chemie und Strukturchemie, Heinrich-Heine-Universität Düsseldorf, 40204 Düsseldorf, Germany

**Keywords:** goldcarbonyl chloride, carbonylchloridogold(I), Au(CO)Cl, gold nanoclusters, metal-organic frameworks, HKUST-1, Cu-BTC, SURMOF

## Abstract

Gas-phase infiltration of the carbonylchloridogold(I), Au(CO)Cl precursor into the pores of HKUST-1 ([Cu_3_(BTC)_2_(H_2_O)_2_], Cu-BTC) SURMOFs (surface-mounted metal-organic frameworks; BTC = benzene-1,3,5-tricarboxylate) leads to Au(CO)Cl decomposition within the MOF through hydrolysis with the aqua ligands on Cu. Small Au_x_ clusters with an average atom number of x ≈ 5 are formed in the medium-sized pores of the HKUST-1 matrix. These gold nanoclusters are homogeneously distributed and crystallographically ordered, which was supported by simulations of the powder X-ray diffractometric characterization. Au_x_@HKUST-1 was further characterized by scanning electron microscopy (SEM) and infrared reflection absorption (IRRA) as well as Raman spectroscopy, time-of-flight secondary ion mass spectrometry (ToF-SIMS), X-ray photoelectron spectroscopy (XPS) and inductively coupled plasma optical emission spectroscopy (ICP-OES).

## 1. Introduction

Metal-organic frameworks (MOFs) [1,2,3,4] are potentially porous and mostly crystalline three-dimensional solids, which attract considerable attention in a wide range of envisioned applications [5], e.g., in catalysis [6], hydrogen storage [7], separations [8], as sensors [9,10,11], for nanocluster formation [12,13], or precursors for electrocatalysts [12,14,15]. Open metal sites (OMSs), also named coordinatively unsaturated metal sites (CUSs), at the metal center possess a crucial role in some applications [16,17,18]. In order to utilize OMSs in MOFs, an activation process is necessary to remove coordinating solvent molecules (e.g., H_2_O or EtOH) from the metal ions and thereby form the open metal sites. The MOF [Cu_3_(BTC)_2_(H_2_O)_2_], also named Cu-BTC, MOF-199, or originally HKUST-1 [19] (Hong Kong University of Science and Technology-1) is constructed from Cu_2_ units coordinated by four 1,3,5-benzenetricarboxylate (BTC) linkers in a paddle-wheel fashion [20,21]. The two axial positions along the Cu_2_ handle contain labile solvent molecules, usually a water ligand from the aqueous synthesis, which can be partially removed due to the Jahn–Teller effect for the Cu(II)-d^9^-ion [18,22]. It is, however, challenging to reach near quantitative removal of the solvent ligand [18,23].

Metal@MOF materials (for metals like Au [24], Zn [25] and Pd [26]) are interesting hybrid materials and a large number of papers on them have been published in the last decade [27]. In previous work, we demonstrated that highly oriented and crystalline MOF coatings, e.g., surface-mounted metal-organic frameworks (SURMOFs) [28,29], can be employed as hosts for the nucleation and growth of small metal and metal oxide clusters [30], e.g., Bi_2_O_3_ [31]. In addition to neutral metal-organic precursors for the formation of metal oxide clusters, various other species, e.g., charged La^3+^ ions, have been successfully loaded into HKUST-1 SURMOF thin film [32].

Gold nanoparticles (Au-NPs) on, or in, HKUST-1 powders have been inter alia prepared for sensing applications [33,34,35,36,37,38], chemo-photothermal therapy [39], or hydrogenation of olefins [40]. For gold nanoparticles inside HKUST-1 (Au-NPs@HKUST-1) the prepared MOF was added to separately prepared Au-NPs [35], HAuCl_4_ reduced with simultaneous formation of HKUST-1 from its precursors [39,40], or HKUST-1 mixed with HAuCl_4_ and the later reduced by sodium citrate [38]. Thereby the nanoparticle size and its uniform distribution inside the MOF cannot be well controlled.

**Figure 1:**
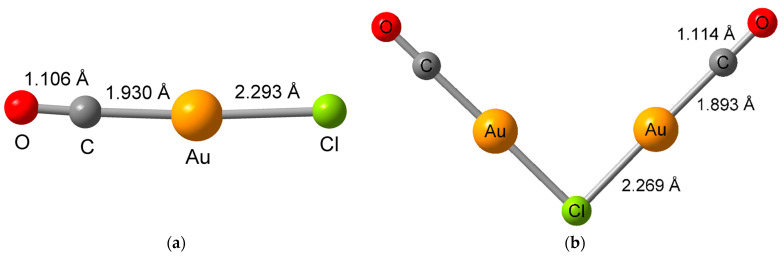
Structure of (**a**) the (OC)Au···Cl moiety in the solid-state adduct [(OC)-Au···ClAl(OR^F^)_3_] and (**b**) the µ-chloridodicarbonyldigold(I) cation in [Au_2_(CO)_2_Cl]^+^[Al(OR^F^)_4_]^−^ (R^F^ = C(CF_3_)_3_) [46]. The overall length in (OC)Au···Cl from O to Cl is ~5.3 Å to which the van der Waals (vdW) radii of O (1.52 Å) and Cl (1.75 Å) would have to be added for the full length of OC-Au-Cl. For the cross-section along the molecular axis twice the vdW radius of Cl can be taken since the vdW radius of Au (1.66 Å) is smaller [47].

In this work, we report a solvent-free gas-phase infiltration process that directly decomposes the carbonylchloridogold(I), Au(CO)Cl, a precursor in an HKUST-1 SURMOF for a highly controlled synthesis of gold nanoclusters inside the MOF. The commercially available Au(CO)Cl has been used for the synthesis of gold nanoparticles in solution [41], albeit not in connection with MOFs. Au(CO)Cl is a volatile organo-gold compound that crystallizes as a colorless solid, being extremely moisture- and temperature-sensitive but stable at room temperature [42,43,44,45]. The solid-state structure of neat Au(CO)Cl is not known but the adduct structure [(OC)-Au···ClAl(OR^F^)_3_] and the structure of the µ-chloridodicarbonyldigold(I) cation in [Au_2_(CO)_2_Cl]^+^[Al(OR^F^)_4_]^−^ (R^F^ = C(CF_3_)_3_) have been reported (Figure 1) [46].

## 2. Results and Discussion

**Figure 2:**
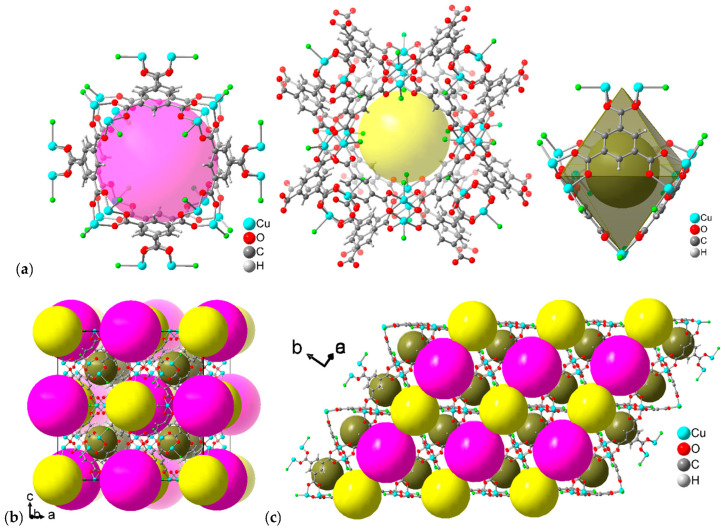
(**a**) Large (pink), medium (yellow) and small (dark yellow) pores in HKUST-1 with the surrounding framework (the three objects are not drawn to scale). The green atoms on Cu depict the aqua ligands. (**b**,**c**) Packing diagrams of HKUST-1 with the three different pores and (**b**) the c axis. i.e., [001] direction vertical, (**c**) the abc diagonal, i.e., [111] direction vertical. Structures were drawn with the program DIAMOND [48] using the cif file with CCDC Refcode FIQCEN [19] from the Cambridge Crystallographic Data Center, CCDC.

The structure of HKUST-1 contains three types of cages: large pores (~11 Å diameter), medium-sized pores (~9 Å) and small pores (~6 Å) (Figure 2a). The pore diameters take into account the van der Waals surface of the surrounding network. The large and medium-sized pores belong to channels that run along the *a*, *b* and *c* axis in the cubic structure of HKUST-1. In each channel direction, the large and medium-sized cavities alternate (Figure 2b). As noted above, each Cu atom binds an aqua ligand at the apical position in the as-synthesized form. The Cu-OH_2_ bond length is slightly elongated (2.165 Å) over the Cu-O (carboxylate) bond (1.952 Å) in the structure of HKUST-1 [19]. Upon removal of the aqua ligand through suitable activation, an open metal site (OMS) (coordinatively unsaturated site, CUS) can form. However, thermal-only activation of HKUST-1 at 70 °C for 12 h under a vacuum of 7 × 10^−4^ bar gave 69% of open metal sites. To reach 93% of OMS a temperature of 230 °C was needed. With a preceding water-to-acetone exchange before thermal activation, 78% OMS could be reached at 50 °C for 12 h, and 98% OMS at 200 °C [23]. Here, the HKUST-1 SURMOF films were activated by ultrasonication in dichloromethane for 5 min and dried using an inert gas flow, followed by heating in the oven at 100 °C for 5 h (no vacuum). This will remove the solvent from the pores but not the Cu-coordinated aqua ligands. It is important to note, that all aqua ligands face toward the open space of the medium-sized pores (yellow sphere in Figure 2a–c).

The HKUST-1 SURMOFs were grown on modified Au substrates or Si-wafer substrates for the ToF-SIMS measurements. The layer-by-layer growth was carried out using the liquid-phase epitaxy (LPE) method [30]. The surface of the gold substrates was modified by the deposition of a self-assembled monolayer (SAM), either from 16-mercaptohexadecanoic acid (MHDA) or from 11-mercapto-1-undecanol (MUD).

**Figure 3:**
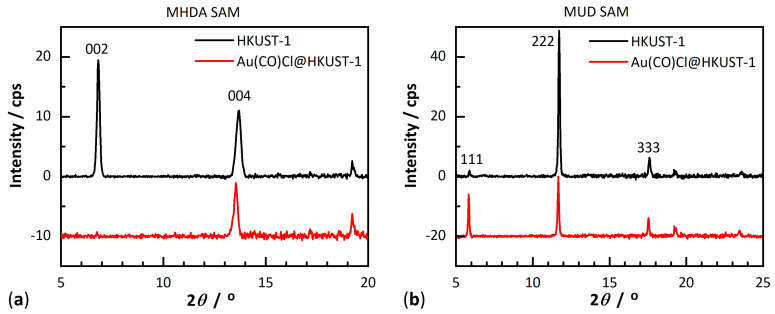
Powder X-ray diffraction (PXRD) patterns recorded for (**a**) empty (black) and after loading Au(CO)Cl (red) in the HKUST-1 SURMOF grown on an MHDA-SAM-modified Au substrate; (**b**) empty (black) and after loading Au(CO)Cl (red) in the HKUST-1 SURMOF grown on a MUD-SAM-modified Au substrate. The reflections are designated by the Miller indices hkl.

Figure 3 shows the powder X-ray diffraction (PXRD) patterns recorded in an out-of-plane geometry for the pristine, monolithic and oriented HKUST-1 SURMOFs. As reported in previous works [30], the 002 and 004 reflections on MHDA-SAM and the 111, as well as the 222 reflection on MUD-SAM, are well-defined and sharp and their positions and relative intensities are in agreement with simulations (Figure S3, Supplementary Materials), indicating a high crystallinity and orientation of the monolithic HKUST-1 thin films. The absence of diffraction peaks for other crystallographic directions in the out-of-plane data reveals that the SURMOFs growth proceeded highly oriented only along the [001] crystallographic direction (Figure 2b) on MHDA-SAM and along the [111] crystallographic direction (Figure 2c) on MUD-SAM. On a Si wafer HKUST-1 grows in a mixed orientation (Figure S4).

The loading of Au(CO)Cl into the pores of the HKUST-1 SURMOFs took place through the gas phase at 80 °C (for details, see Experimental section; Figure S2). Au(CO)Cl sublimes at about 75 °C [42]. At 110 °C concomitant decomposition occurs according to 2Au(CO)Cl → 2Au + CO + COCl_2_. With water, the gold(carbonyl)chloride decomposes according to 2Au(CO)Cl + H_2_O → 2Au + CO + CO_2_ + 2HCl [43]. This would also include reactions with the aqua ligands on the apical Cu coordination sites.

PXRD data recorded after loading Au(CO)Cl into the HKUST-1 SURMOF (Figure 3, red curves) confirmed the retention of the crystallinity of the host matrix and no apparent additional diffraction peaks. However, after loading Au(CO)Cl into HKUST-1, the ratio of the diffraction peak intensities significantly changed. The 002/004 reflection ratio (out-of-plane data) from the MHDA-SAM sample significantly decreased from 1.76 for the pristine HKUST-1 SURMOF to near zero for the Au(CO)Cl-loaded thin films (Figure 3a). In addition, the in-plane PXRD data showed that the 200/400 ratio of reflection intensities dropped from 1.32 to near zero (Figure S5). For the MUD-SAM sample, the out-of-plane PXRD data also revealed retention of crystallinity with pronounced changes: the reflection ratio of 111/222 increased from 0.04 to 0.70 (Figure 3b). For the mixed orientation of HKUST-1 on a Si wafer, the same changes in relative intensities are observed upon loading with Au(CO)Cl, that is the 002/004 ratio decreased to near zero and the 111/222 ratio increased from near zero to about 0.55 (Figure S4). Longer loading times did not lead to a further change in the relative PXRD peak intensities. This change in relative PXRD peak intensities demonstrates that Au(CO)Cl (or its decomposition product, see below) was loaded into the pores of the HKUST-1 SURMOF.

The same loading process has been carried out for HKUST-1 powder for comparison. The diffractogram of the empty HKUST-1 powder matches the simulation (Figure S6). The changes in relative peak intensities observed for the powder proceeded in the same direction albeit less pronounced than those observed in the HKUST-1 SURMOF thin films (Figure 3). The reflection ratio of 002/004 decreased after the loading of Au(CO)Cl and the reflection ratio of 111/222 increased. The less pronounced change in the peak intensities in the loaded powder is explained by an incomplete loading because of longer diffusion path lengths in the larger powder particles together with pore-blocking when compared to the only ~200 nm thin films.

**Figure 4:**
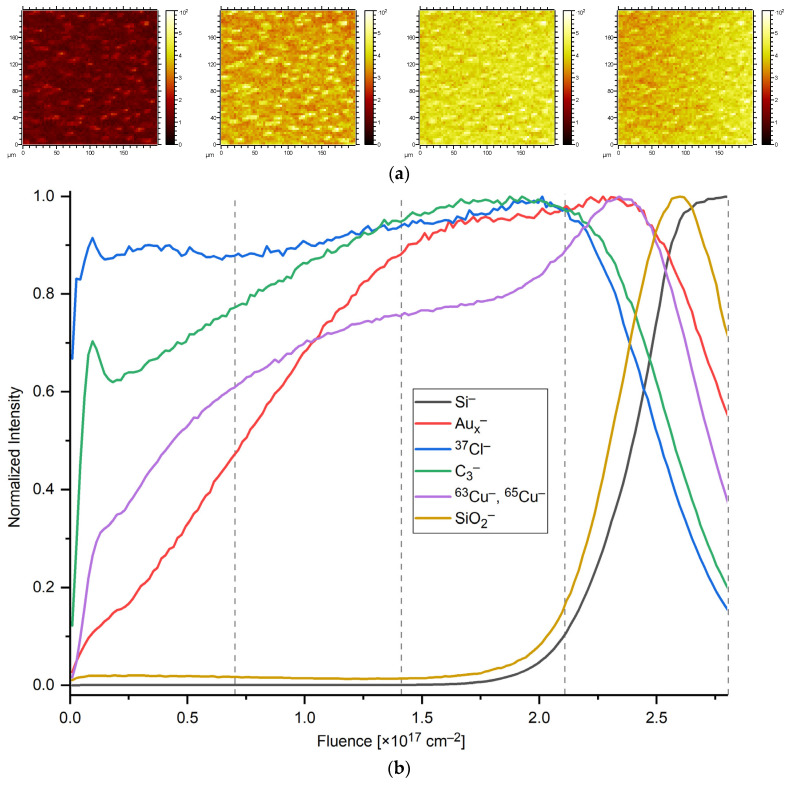
Time-of-flight secondary ion mass spectrometry (ToF-SIMS) depth profiling. (**a**) Lateral distribution of gold (sum of Au_x_^−^ signals, x = 1…5) from each quarter of the analytical run (dashed grey lines) corresponding to the top to bottom of the layer, left to right). (**b**) Signal intensities of silicon (black line), SiO_2_ (yellow line) the sum of both copper isotopes (violet line), ^37^Cl^−^ (blue line), C_3_^−^ (green line), and the sum of Au_x_^−^ species (x = 1…5) (red line) plotted against sputter ion fluence as an arbitrary measure for depth. All signals are scaled to 1 for their respective maximum intensity.

Cross-sections of Au(CO)Cl-loaded and non-loaded HKUST-1 thin films (~45 layers of Au(CO)Cl@HKUST-1) reveal significant electron contrast of the Au-loaded layers (Figure S1). To analyze the lateral (Figure 4a) and depth distribution of gold (Figure 4b) loaded into an HKUST-1 sample (50 cycles, prepared on Si), loaded with Au(CO)Cl and kept in ambient air for 3 days, was subjected to ToF-SIMS dual beam depth profiling. Figure 4 shows the signal intensities of silicon (Si^−^, substrate material), the sum of both copper isotopes (^63^Cu^−^ and ^65^Cu^−^), ^37^Cl^−^ (24% natural abundance), C_3_^−^ (from the organic linker), and the sum of Au and gold clusters (Au_2_ to Au_5_) as Au_x_^−^, obtained during the depth profiling of the sample. All signals are scaled to 1 for maximum intensity. Mixed metal clusters like CuAu_2_^−^ and Cu_2_Au^−^ were also detected.

While gold, like a few other noble metals, tends to yield strong signals in negative secondary ion polarity, copper usually exhibits stronger Cu^+^ secondary ion signals. In this study, Cs^+^ ion sputter bombardment was applied for sample erosion to increase the yield of negatively charged secondary ions by the implantation of Cs into the sample. As shown in Figure 4b, the intensities of chlorine and carbon ions rise immediately after the onset of erosion due to the removal of some airborne surface contaminations and the establishment of sputter equilibrium. In addition, the copper ion signal intensity increases rapidly during sample erosion, whereas gold intensities are increasing more slowly because of the degraded surface layer (cf. Figure S1). When the silicon substrate is reached, at a fluence of approximately 2.5 × 10^17^ ions/cm^2^, the signal intensities from the SURMOF, chlorine ion and Au species drop, and the SiO_2_^−^ from the native oxide layer on the silicon wafer and finally the Si^−^ signal from the bulk rises. In addition to this obvious difference in their depth distribution, the lateral distributions of gold and copper are also quite different, especially in the topmost layer of the SURMOF. While copper is homogeneously distributed in the SURMOF volume, the distribution of gold is quite non-uniform in the surface regions of the HKUST-1 layer as shown by the lateral distribution maps given in Figure 4a above their respective depth zones. Especially in the topmost layers of the sample, individual spots of higher local gold intensities are found. Another representation of these data, showing the structure of copper and gold iso-surfaces (surfaces of constant signal levels) is given in Figure S8.

The relative Intensities of detected Au_x_^−^ species changed during sputter erosion (Figure S7). At low Cs ion fluence of less than 0.1 × 10^17^ ions/cm^2^ the intensity of the Au_x_^−^ clusters Au_3_^−^, Au_2_^−^ and Au_5_^−^ normalized to Au_1_^−^ is highest and their intensity drops to a rather steady level at a primary ion fluence of ~0.5 × 10^17^ ions/cm^2^ (Figure S7). The order of cluster intensity with Au_3_^−^ > Au_2_^−^ > Au_5_^−^ remained the same up to a fluence of 2.5 × 10^17^ ions/cm^2^, which is common for metallic bulk gold. Overall gold clusters up to Au_7_^−^ were detected (limited by the SIMS analysis cycle time cutting the mass range). The formation of gold clusters can be attributed to a decomposition of the Au(CO)Cl precursor in HKUST-1, yielding Au_x_ clusters inside HKUST-1 as Au_x_@HKUST-1 (see below). A drop in normalized (to Au_1_^−^) intensities of the Au_x_^−^ clusters (x = 3, 2, 5) was observed for the lowest sputter fluences. It is unclear if the decrease in larger gold cluster contributions with increasing depth is due to cluster fragmentation upon continuous sputtering or if the larger clusters Au_3_^−^ and Au_5_^−^ have preferentially formed near the HKUST-1 surface.

Table S1 shows the observed intensity distribution of gold cluster secondary ions under erosion conditions for gold nanoparticles in the SURMOF and for a metallic bulk gold sample. In both cases, the bombardment with primary and sputter ions liberates a series of small gold clusters and monoatomic gold ions. This distribution indicates a preference for uneven gold atom numbers in the emitted clusters. This is not the true size distribution in the sample in either Au_x_@HKUST-1 or bulk gold. Rather the distribution is the result of a harsh ion bombardment of the sample followed by a characteristic ionization process. Under the harsh ion bombardment, especially with monoatomic Cs ions used for erosion, this leads to some amorphization of the topmost layer of a specimen, the gold clusters in Au_x_@HKUST-1 will fragment and the fragments can also recombine, thereby giving a series of Au_x_ signals (x = 2–7). Furthermore, this in situ amorphization also leads to the formation of mixed-metal or “alloy” clusters CuAu_2_^−^ and Cu_2_Au^−^, which are not present in the Au_x_@HKUST-1 sample. Thus, the sequence and intensity of observed gold clusters have nothing in common with the size distribution in the sample itself. It is important to note that the observed Au_x_ signal ratio shows a strong resemblance to that of metallic bulk gold (Table S1), and not only individual Au ions as would be expected from the initial precursor. Furthermore, it is evident that the distribution of the emitted gold clusters from the Au_x_@HKUST-1 sample shows in comparison to bulk gold a shift to lower gold atom numbers. Hence, the true gold cluster size and size distribution within the SURMOF matrix cannot be determined by ToF-SIMS.

Inductively coupled plasma optical emission spectrometry (ICP-OES) on a Au(CO)Cl@HKUST-1 powder sample (Figure S9) yielded a Cu concentration of 3.6 mg/L and a Au concentration of 1.4 mg/L in the solution obtained from acidic digestion, corresponding to 0.057 mmol/L and 0.007 mmol/L, respectively. From this, a molar Cu:Au ratio of ~8:1 is derived. An X-ray photoelectron spectroscopic (XPS) analysis (Figure S10) of a Au(CO)Cl@HKUST-1 SURMOF sample gave a Cu:Au ratio of ~6:1. With 48 Cu atoms in the unit cell of HKUST-1, this would mean between 6 to 8 Au atoms per unit cell. For powders, less Au(CO)Cl may have been incorporated because of longer diffusion path lengths in the larger powder particles together with pore-blocking compared to the only ~200 nm thin SURMOF films

**Figure 5:**
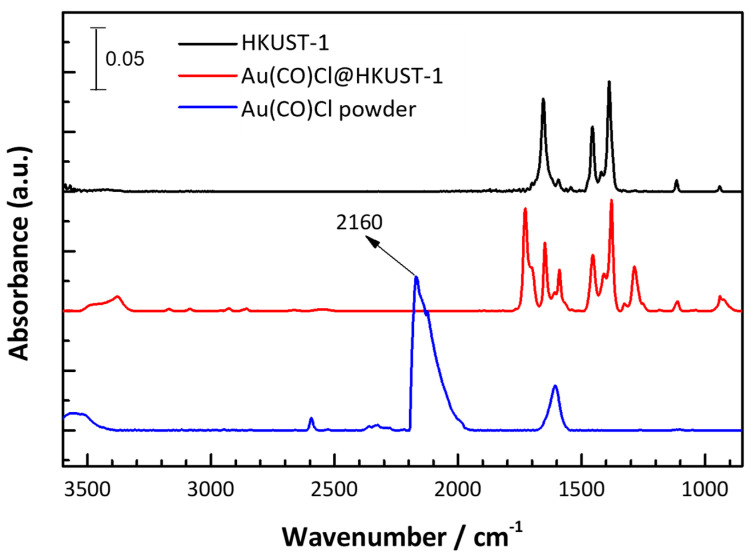
IR spectrum at 20 °C for empty HKUST-1 (black), for Au(CO)Cl@HKUST-1 (red) and Au(CO)Cl powder (blue).

In order to further characterize the state of the gold guest species from the loading of Au(CO)Cl in the pores of HKUST-1, we have carried out investigations using infrared reflection absorption spectroscopy (IRRAS) and Raman spectroscopy under ambient conditions. The Au(CO)Cl molecule has a distinct C≡O stretching vibration at 2160 cm^−1^ (Figure 5).

The IRRAS measurement for HKUST-1 shows broad and strong bands at 1655 cm^−1^ (COO^−^ asymmetric stretching), 1457 cm^−1^ (COO^–^ symmetric stretching) and 1388 cm^−1^ (COO^−^ symmetric stretching) (Figure 5, black spectrum). Bands at 1110 cm^−1^ and 940 cm^−1^ are assigned to δC-H vibrations [23]. After loading of Au(CO)Cl into the porous HKUST-1 SURMOF, two new peaks were observed, with a characteristic C=O stretching vibration at 1728 cm^−1^ and C-O stretching vibration at 1286 cm^−1^ (Figure 5, red spectrum). These bands are very close to the C=O and C–OH stretching vibrations of free (protonated) carboxylic acid groups [23,49,50]. Therefore, we believe that Au(CO)Cl has reacted with the axial aqua ligands at Cu under the formation of gold atoms, which then form small clusters, CO, CO_2_ and HCl: 2Au(CO)Cl + 2H_2_O → 2Au + CO + CO_2_ + 2HCl [43]. The hydrogen chloride can then leave the framework or protonate part of the carboxylate groups, forming -COOH which may even remain partially bound to the Cu_2_ pair of the SBU in HKUST-1. The chloride ion from HCl can either coordinate to Cu at the labile apical position or occupy the former carboxylate coordination site. Note that ToF-SIMS analysis had shown the release of chloride (Figure 4b) since Au and Cl signals are not correlated in depth.

At 293 K no CO adsorption is seen in Au(CO)Cl@HKUST-1 SURMOF by IR in agreement with the literature where such adsorption is described at 100 K in Cu-basolite C300 (Cu-BTC) [49] or at 295 K on Cu+ sites present in Cu-basolite [50]. IR Bands for CO adsorbed at 100 K on activated Cu-basolite appear between 2212 and 2123 cm^−1^ with the most intense CO bands around 2170 cm^−1^ [49]. For CO adsorbed at 295 K in Cu-basolite the prominent band is recorded at 2127 cm^−1^ and assigned to CO adsorbed on Cu^+^ [50]. The presence of Cu+ in HKUST-1 could be due to Cu(I) oxide impurities or due to the reduction of Cu^2+^ in HKUST-1 by CO, with the latter Cu^+^ introduction by redox treatments with reducing gases being the preferred interpretation [50].

**Figure 6:**
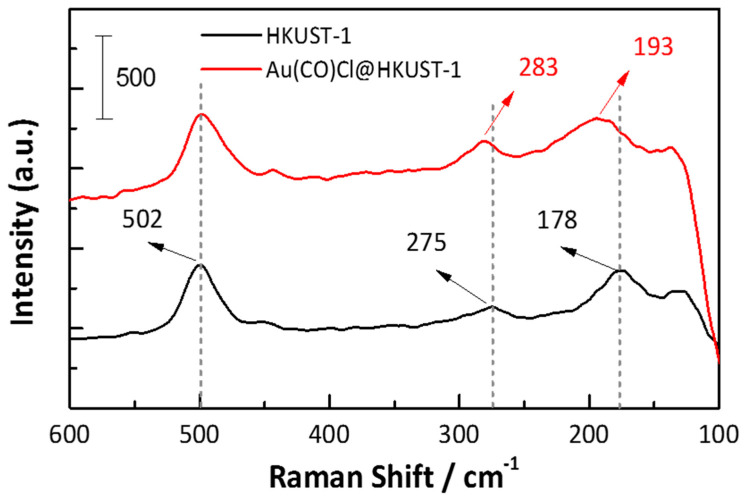
Raman spectra of empty HKUST-1 (black) and Au(CO)Cl@HKUST-1 SURMOFs (red). The dip of the Raman spectrum of Au(CO)Cl@HKUST-1 at the low-frequency end is due to a broad-band luminescence, which is known for small Au_x_ nanoclusters [53,54], still remaining after the subtraction of the baseline from the background spectra.

The Raman spectrum in Figure 6 exhibits vibrational modes in the low-frequency region involving Cu^2+^ species, especially a stretching vibration mode of Cu···Cu (actually H_2_O-Cu···Cu-OH_2_) (at 178 cm^−1^), for Cu-O(aqua), with O(aqua) being the oxygen atom of the weakly coordinated water molecule on the apical position (275 cm^−1^) and Cu-O bonding to oxygen atoms of the carboxylate bridges (at approximately 502 cm^−1^) in pristine HKUST-1 (Figure 6, black spectrum) which agrees well with literature assignments reported for HKUST-1 [51,52] Upon loading with Au(CO)Cl and subsequent reaction, as discussed above, the Raman bands of the Cu···Cu unit shift to approximately 193 cm^−1^ and 283 cm^−1^ (Figure 6 red curve).

Removing the aqua ligands upon reaction with Au(CO)Cl will lower the mass at the Cu atoms and, thereby, induce the detected shifts to higher energies for Cu···Cu. Thereby we note a broadening of the band, indicative of a range of Cu···Cu modes and fragments. The increase in wavenumber for the band at 275 cm^−1^ is reasoned by replacing in part the weakly coordinated aqua ligand with a more strongly coordinated anionic chloride ligand. We note, however, that a more recent theoretical and experimental study [55] assigned the mode at 170 cm^−1^ to a reticular vibration involving the out-of-plane deformation of the benzene ring and the ring CO_2_{Cu_2_}O_2_C formed by Cu_2_ and two carboxylate groups. The Cu···Cu stretch was calculated at 210 cm^−1^, albeit with a negligible intensity so as to remain undetected experimentally. The peak at 273 cm^−1^ was assigned to O–Cu–O bending. The band at 498 cm^−1^ was assigned to Cu–O stretching vibrations, which result in an in-phase breathing motion of two interconnected CO_2_{Cu_2_}O_2_C rings, i.e., the paddlewheel unit [55].

**Figure 7:**
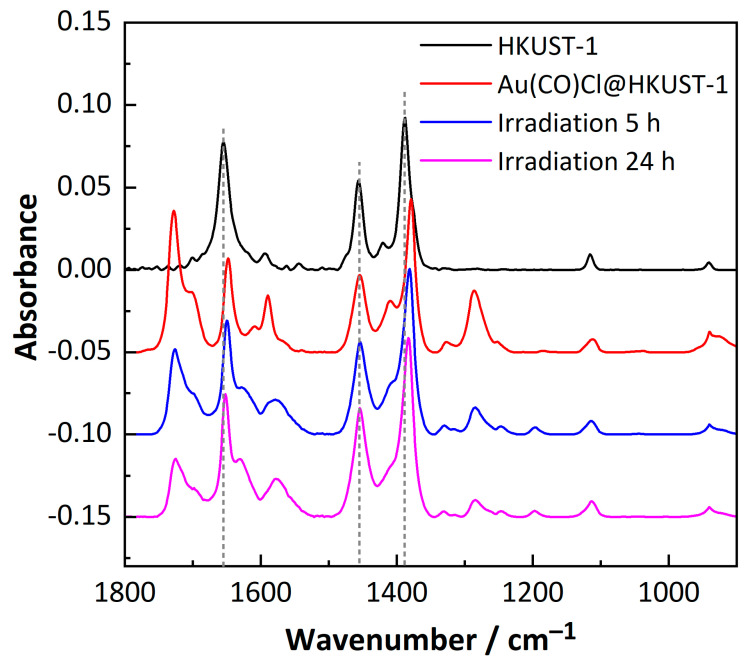
IR spectra for empty HKUST-1 SURMOF (black), after loading with Au(CO)Cl (red) and exposed to UV light for 5 h (blue) and exposed to UV light for 24 h (magenta).

The stability of Au(CO)Cl@HKUST-1 with respect to the reaction products was investigated through a UV irradiation experiment and followed with IRRAS over time (Figure 7). The Au(CO)Cl@HKUST-1 sample was exposed to 365 nm UV light for 5 h or 24 h. With increasing time for the UV irradiation, the C=O stretching vibration at 1728 cm^−1^ and C-O stretching vibration at 1286 cm^−1^, which had appeared upon Au(CO)Cl loading, decrease again in intensity. These bands were assigned to the C=O and C–OH stretching vibrations of free (protonated) carboxylic acid groups [23,49,50]. Thus, UV irradiation appears to enforce the deprotonation with release of the HCl from the HKUST-1 SURMOF.

**Figure 8:**
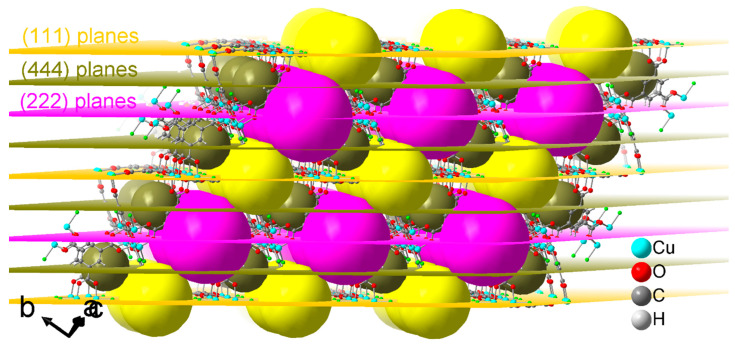
Position of the set of (111), (222) and (444) planes in HKUST-1 (cif file with Refcode FIQCEN (axis a = 26.343(5) Å) [19] from the CCDC). The spacing of the set of (111) planes is 15.2 Å, and the spacing of the set of (222) planes, which also includes the set of (111) planes, is 7.6 Å. The spacing of the set of (444) planes (including (111) and (222) planes) is 3.8 Å. For the description of the different pores see Figure 2a–c and the accompanying text.

**Figure 9:**
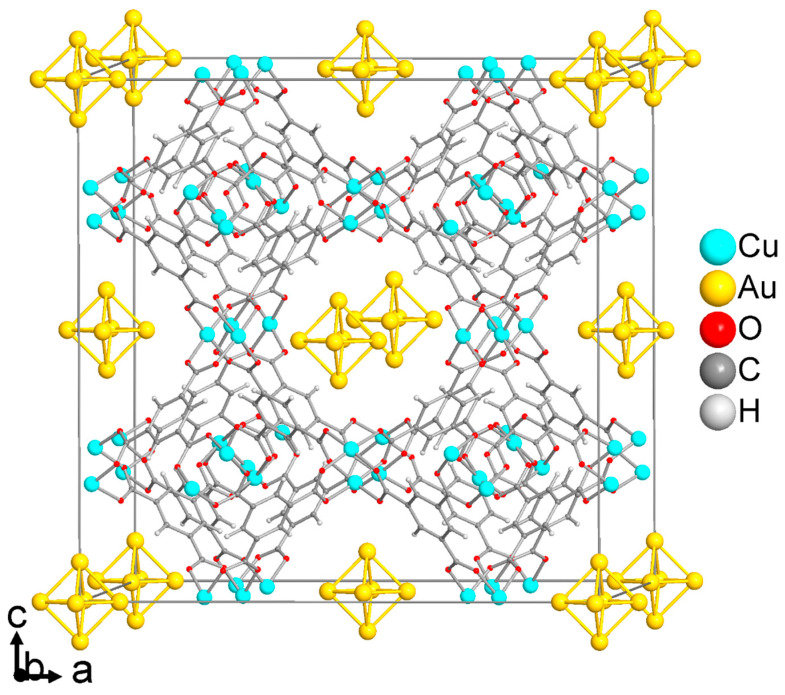
Section of the packing diagram of HKUST-1 with Au clusters in the medium-sized pores. The appearance of an ideal octahedral Au cluster is an artifact from the cubic symmetry where a single Au atom placed off-center in the pore will be positioned at the vertices of an octahedron by symmetry. The Au···Au separation along the edges of the octahedron was set to 2.8 Å. The electron density of the Au cluster was adjusted to correspond to five Au atoms in the pore. For the illustration, the solvent-free structure of HKUST-1 with CSD-Refcode DOTSOV, CCDC 697917 [55], was used.

**Figure 10:**
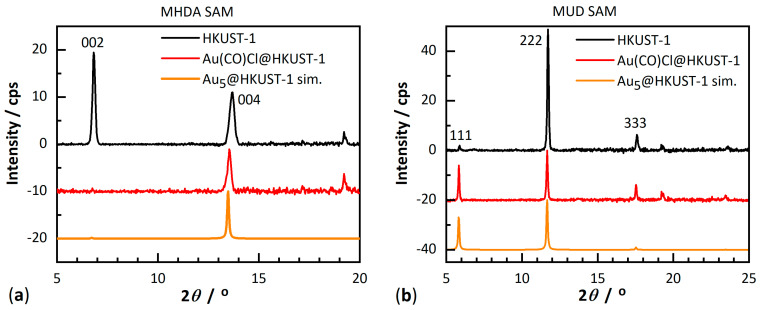
Experimental powder X-ray diffraction (PXRD) patterns for empty HKUST-1 SURMOF (black) and after loading Au(CO)Cl (red) into the SURMOF which was (**a**) grown on an MHDA-SAM modified Au substrate and (**b**) grown on a MUD-SAM modified Au substrate (cf. Figure 3). The yellow diffractograms are the simulation for Au_5_@HKUST-1 SURMOF with preferred [001] (**a**) and [111] (**b**) orientation. The simulated PXRD patterns have been calculated with the program MERCURY [56] using the cif file for HKUST-1 with CSD-Refcode DOTSOV, CCDC 697917 [57] and setting the March–Dollase parameter to 0.01. The reflections are designated by the Miller indices hkl.

From the above analyses, it became evident that Au(CO)Cl decomposes within the pores of the HKUST-1 SURMOF with a reduction to gold atoms or small clusters. The formation of small gold clusters Au_x_ is further supported by a simulation of the diffractograms for Au(CO)Cl@HKUST-1 in Figure 3 (red curves). The features that had to be reproduced were the near zero ratio for the 002/004 reflection—i.e., an almost absence of the 002 reflection—in the MHDA-SAM sample with HKUST-1 grown along the [001] direction (Figure 3a) and an increase in the intensity of the 111 reflection to a ratio of about 0.7 for 111/222 in the MUD-SAM sample where HKUST-1 was grown along the [111] direction (Figure 3b). There are three different pores in HKUST-1 in which the gold clusters could form (Figure 2). The key to where the gold clusters form comes from the change in the 111 reflection. According to the experimental data, the 111 reflection is very weak in the pristine HKUST-1, both in the SURMOF (Figure 3, Figures S4 and S5) and in the powder (Figure S6). The increase in 111 reflection intensity must be caused by the location/arrangement of the Au clusters within the unit cell of HKUST-1 at a spacing that is equal to the spacing of the (111) reflective planes (lattice planes) of HKUST-1 (Figure 8) (see Section S1 under powder X-ray diffraction (PXRD) powders). Figure 8 illustrates the position of the set of (111), (222) and (444) planes in HKUST-1 and it is evident that the set of (111) planes pass through the medium-sized (yellow) pores. Thus, the gold clusters seem to form in these pores. The medium-sized pore has the aqua ligands on the apical copper site pointing into its open space (Figure 2b) and most likely the reaction of Au(CO)Cl with these aqua ligands according to 2Au(CO)Cl + H_2_O → 2Au + CO + CO_2_ + 2HCl [43] forms gold atoms, which then assemble into small clusters. A simulation of an HKUST-1 structure with the electron density of an Au_5_ cluster in the medium-sized pore (Figure 9) reproduces the experimental PXRD patterns after loading Au(CO)Cl in the HKUST-1 SURMOFs grown on an MHDA-SAM and a MUD-SAM modified Au substrate (Figure 10, cf. Figure 3). Simulating the diffractograms with an Au atom only in the large (pink) pore will give a strong 002 reflection. In addition, when only one Au atom is placed in the center of the medium-sized pore or one Au atom each in both the large and medium-sized pores, the 002 reflections remain stronger than the 004 reflection for the preferred [001] orientation. Thus, the simulation of the experimental diffractograms for Au(CO)Cl@HKUST-1 in both preferred orientations along [001] and [111] requires the assumption of Au_x_ clusters with an average of x ≈ 5 almost exclusively in the medium-sized pores.

## 3. Materials and Methods

### 3.1. Chemicals and Equipment

Chemicals from commercial suppliers were used as received. 16-mercaptohexadecanoic acid (MHDA, 99%) and 11-mercapto-1-undecanol (MUD, 99%) were purchased from Aldrich. Copper acetate di-hydrate (Cu_2_(O_2_CCH_3_)_4_·2H_2_O) (p.a. quality) was purchased from Sigma-Aldrich (St. Louis, MO, USA). Benzene-1,3,5-tricarboxylic acid (H_3_BTC), ethanol, dichloromethane and dichloroethane were purchased from Sigma-Aldrich, all as p.a. quality. Carbonylchloridogold(I) and Au(CO)Cl (98.5% purity) were purchased from Strem. Nitric acid (65 wt%, p.a. quality) was purchased from Sigma-Aldrich.

X-ray diffraction (XRD) of the thin films was done by using a Bruker D8 Advance diffractometer (Bruker, Ettlingen/Karlsruhe, Germany) equipped with a Si-strip detector (Lynxeye position sensitive detector) with Cu K_α1,2_ radiation (λ = 1.5418 Å) in θ–θ geometry, variable slit on primary circle. Scans were run over various ranges with a step width of 0.024° 2θ and 84 s, or, for higher order peaks, up to 336 s per step. The 2θ angle scanning range was 5° to 60°. In-plane XRD was conducted using a Bruker D8 Discover equipped with a quarter Eulerian cradle, tilt-stage, and 2.3° Soller-slits installed on both sides. A Göbel-mirror and a Lynxeye position-sensitive detector in θ–2θ geometry were applied.

X-ray diffraction (XRD) patterns of powders were measured on a Bruker D8 Advance diffractometer equipped with a Lynxeye position sensitive detector, a variable divergence slit using Cu K_α1,2_ radiation over a 2θ scan range of 2° to 80°, a step width of 0.020° and a total counting time of 3400 s (4 repetitions with 850 s each). The sample was rotated during measurement.

Infrared reflection absorption (IRRA) spectroscopy: all samples were recorded using an FTIR spectrometer (Bruker VERTEX 80, Bruker, Ettlingen/Karlsruhe, Germany) with a resolution of 2 cm^−1^ at an incidence angle of 80° relative to the surface normal. Liquid nitrogen is used to cool the mercury cadmium telluride (MCT) narrow band (4000–400 cm^−1^) detector. Dry air was purged continuously through the spectrometer and sample compartment, which reduces the possibility of atmospheric water or CO_2_ contamination on the samples. Samples were measured until the water absorption bands from ambient air disappeared (900–1300 scans). The data were processed using the Bruker OPUS^®^ software version 7.2. Perdeuterated hexadecane thiol SAMs on Au/Ti/Silicon substrates were used for reference measurements.

Scanning electron microscopy (SEM): HR-SEM cross-sectional measurements have been performed on a Zeiss HR-SEM (Gemini Class, Zeiss, Oberkochen, Germany) at 3–5 kV.

Time-of-flight secondary ion mass spectrometry (ToF-SIMS): ToF-SIMS was performed on a TOF-SIMS5 instrument (ION-TOF GmbH, Münster, Germany). This spectrometer is equipped with a Bi cluster primary ion source and a reflectron-type time-of-flight analyzer. The ultra-high vacuum (UHV) base pressure was below 9 × 10^−9^ mbar. For high mass resolution, the Bi source was operated in the “high current bunched” mode providing short Bi_3_^+^ primary ion pulses at 25 keV energy, a lateral resolution of approx. 4 μm, a target current of 0.4 pA, and a pulse width of 0.95 ns. Spectra were calibrated on the omnipresent C^−^, C_2_^−^ and C_3_^−^ peaks. For depth profiling a dual beam analysis was performed in full-interlaced mode: the primary ion source scanned a field of view of 200 × 200 µm^2^ (64 × 64 data points) and a sputter gun (operated with Cs^+^ ions, 500 eV, scanned over a concentric field of 400 × 400 µm^2^, target current 27 nA) was applied to erode the sample. Thereby, the sputter ion dose density was >18 × 10^3^ times higher than the Bi ion dose density to reduce intermixing during ion bombardment. ToF-SIMS data of this study will be made available at DOI: 10.35097/937 under a CC BY 4.0 license.

Inductively coupled plasma optical emission spectrometry (ICP-OES) was performed using a Perkin-Elmer model Optima 8300 DV equipped with a GemTipTM crossflow nebulizer (Perkin Elmer, Waltham, MA, USA, USA). Au(CO)Cl@HKUST-1 powder samples were dissolved with 65 wt% nitric acid to give a sample concentration within the calibration limits.

X-ray photoelectron spectroscopy (XPS) XPS analyses were performed with an XPS/AES/UPS system with a hemispherical analyzer R4000 of VG Scienta Ltd., St Leonards-on-Sea, UK under ultra-high vacuum conditions (10^−10^ mbar) using an Al K_α1,2_ (1486.3 eV) X-ray radiation source. Spectra were recorded with a pass energy of 200 eV for survey spectra and 100 eV for detailed high-resolution spectra. The step width and the dwell time per step were defined as 0.05 eV and 100 ms, respectively. To compensate for charging, a flood gun with an electron energy of 2 eV was used. The deconvolution of peaks was done using CASA XPS software, in which peaks were fitted using Shirley background, Gaussian/Lorentzian (GL) line shapes and a Marquardt-Levenberg optimization algorithm.

### 3.2. Fabrication of HKUST-1 SURMOFs

All HKUST-1 SURMOFs used in this work were grown using the liquid-phase epitaxy (LPE) method as described in detail in a previous publication [30], either on modified Au substrates or on Si-wafer substrates for the ToF-SIMS measurements. The surface modification of gold substrates was carried out by depositing a self-assembled monolayer (SAM) made from 16-mercaptohexadecanoic acid (MHDA) or 11-mercapto-1-undecanol (MUD). The MOF precursors were then iteratively hand-sprayed onto the substrate using the layer-by-layer (LBL) LPE technique. The spray times were 15 s for the ethanolic copper acetate solution (0.3 mmol L^−1^) and 25 s for the ethanolic benzene-1,3,5-tricarboxylic acid (H_3_BTC) solution (0.15 mmol L^−1^). Each spray step was followed by a rinsing step (3 s) with pure ethanol to remove residual reactants. A total of about 20–50 growth cycles were used for all SURMOFs investigated in this work. We define a single LBL cycle as both the application of a single round of the metal and linker constituents. Thus, one cycle corresponds to one layer. Before further processing, all SURMOF samples were soaked in dichloromethane for 5 min under sonication, dried using an inert gas flow, then heated in the oven at 100 °C for 5 h and were verified by powder X-ray diffraction (PXRD). Cross-sectional images recorded by scanning electron microscopy (SEM) demonstrate that the thickness of 45 layers SURMOFs was about 200 nm (Figure S1).

### 3.3. Fabrication of HKUST-1 Powder

The HKUST-1 powder samples were prepared as reported elsewhere [19]. The solutions of prior dissolved copper nitrate trihydrate (1.8 mmol L^−1^, 0.435 g), and trimesic acid (1 mmol/L, 0.241 g) in de-ionized water and ethanol respectively were mixed and transferred to a 50 mL Teflon inlet inserted in a stainless-steel autoclave. The autoclave was heated for 12 h at 180 °C. After cooling, the product was separated using a centrifuge at 5000 rpm, washed with ethanol three times and dried in an oven at 90 °C for 4 h.

### 3.4. Gas-Phase Loading of Au(CO)Cl into HKUST-1 SURMOFs

Activated HKUST-1 SURMOF thin films were placed into a 250 mL round-bottom flask, together with 10 mg Au(CO)Cl powder which was heated up to 80 °C for 12 h (Figure S2).

## 4. Conclusions

Here we report a synthesis strategy to upload Au(CO)Cl in HKUST-1 as SURMOF or powder for the formation of gold clusters inside the MOF. Au-nanoclusters@HKUST-1 have been successfully prepared by gas-phase infiltration of the molecular Au-cluster precursor Au(CO)Cl into HKUST-1. The homogenous formation and distribution of small Au_x_ clusters within the HKUST-1 pore matrix with an average atom number of x ≈ 5 in the medium-sized pores of HKUST-1 was supported by the simulation of the experimental powder X-ray diffraction patterns of the SURMOFs. The decomposition of the labile and water-sensitive Au(CO)Cl precursor upon reaction with the Cu aqua ligands or adsorbed water in the MOF into gold clusters was further supported by time-of-flight secondary ion mass spectrometry (TOF-SIMS) where Au_x_ clusters up to x = 7 could be detected as well as by IRRA spectroscopy (absence of a CO band). The Au-nanoclusters@HKUST-1 have been further characterized by scanning electron microscopy (SEM), Raman spectroscopy, X-ray photoelectron spectroscopy (XPS) and inductively coupled plasma optical emission spectroscopy (ICP-OES) measurements. In our laboratories further loading processes for different MOF materials either from the gas or liquid phase are currently under investigation.

## Data Availability

ToF-SIMS data of this study will be made available at DOI:10.35097/937 under a CC BY 4.0 license. Other data presented in this study are available upon request from the corresponding authors.

## Supplementary Materials

The following supporting information can be downloaded at https://www.mdpi.com/article/10.3390/molecules28062716/s1. Section S1: Methods; Section S2: SEM image of Au(CO)Cl@HKUST-1 SURMOF (Figure S1); Section S3: Gas-phase loading of Au(CO)Cl into HKUST-1 SURMOFs (Figure S2); Section S4: Powder X-ray diffraction (Figure S3–S6); Section S5: Time-of-flight secondary ion mass spectrometry and iso-surface (Figures S7 and S8, Table S1); Section S6: ICP-OES and XPS (Figures S9 and S10). References [19,56,57,58,59,60,61,62,63,64] are cited in the supplementary materials.

## Sample Availability

Samples of the compounds will not be available.
