# Supplementary material for: Formation of Gold Nanoclusters from Goldcarbonyl Chloride inside the Metal-Organic Framework HKUST-1
Source: Molecules. 2023 Mar 17;28(6):2716. doi: 10.3390/molecules28062716 (PMC10051452; doi:10.3390/molecules28062716)
Supplement: Supplementary file 1 [file molecules-28-02716-s001.zip › molecules-2284110-supplementary.pdf]

Supplementary Material

Formation of Gold Nanoclusters from Goldcarbonyl Chloride inside the Metal-Organic Framework HKUST-1

Zeinab Mohamed Hassan [1,†], Wei Guo [1,†], Alexander Welle [1,2], Robert Oestreich [3], Christoph Janiak [3,*] and Engelbert Redel [1,*]

[1] Karlsruhe Institute of Technology, Institute of Functional Interfaces (IFG), Hermann-von-Helmholtz-Platz 1, 76344 Eggenstein-Leopoldshafen, Germany
[2] Karlsruhe Institute of Technology, Karlsruhe Nano Micro Facility (KNMF), Hermann-von-Helmholtz-Platz 1, 76344 Eggenstein-Leopoldshafen, Germany
[3] Institut für Anorganische Chemie und Strukturchemie, Heinrich-Heine-Universität Düsseldorf, 40204 Düsseldorf, Germany
* Correspondence: janiak@uni-duesseldorf.de (C.J.); engelbert.redel@partner.kit.edu (E.R.)
† These authors contributed equally to this work.

Content:

S1 **Methods** S2

S2 **SEM image of Au(CO)Cl@HKUST-1 SURMOF (Figure S1)** S5

S3 **Gas-phase loading of Au(CO)Cl into HKUST-1 SURMOFs (Figure S2)** S5

S4 **Powder X-ray diffraction (Figure S3 to Figure S6)** S6

S5 **Time-of-flight secondary ion mass spectrometry and iso-surface (Figure S7, Figure S8, Table S1)** S9

S6 **ICP-OES and XPS (Figure S9 and Figure S10)** S10

S1 Methods

Powder X-ray diffraction (PXRD) of thin films: Each sample was characterized by using a Bruker D8 Advance equipped with a Si-strip detector (PSD Lynxeye©; position sensitive detector) with Cu Kα1,2 radiation (λ = 0.15418 nm) in θ–θ geometry, variable slit on primary circle. Scans were run over various ranges with step width of 0.024° 2θ and 84 seconds, for higher order peaks up to 336 seconds per step. The 2θ angle scanning range to observe corresponding peak to deposited film is picked up from 5° to 60°. In-plane XRD was conducted using a Bruker D8 Discover equipped with a quarter Eulerian cradle, tilt-stage, and 2.3° Soller-slits installed on both sides. A Göbel-mirror and a PSD Lynxeye© in θ–2θ geometry were applied.

Powder X-ray diffraction (PXRD) of powders: The empty and loaded HKUST-1 powders were measured on a Bruker D8 Advance equipped with a Lynxeye PSD (position sensitive detector), a variable divergence slit using Cu Kα1,2 radiation over a scan range from 2°2θ to 80°2θ and a step width of 0.020°2θ and a total counting time of 3400 seconds (4 repetitions with 850 seconds each). The sample was rotated during measurement.

In order to understand the experimental data and to further obtain detailed information regarding the distinct positions of the loaded species within the MOF pores, we need to further evaluate the crystal structure factors of the pristine and the Au(CO)Cl loaded HKUST-1 SURMOF. As reported in previous work, the structure factor $F_{(hkl)}$ is an important parameter to understand the relationship between the crystal structure and the intensity of the diffracted X-rays from each crystallographic plane measured [58]. The square of the structure factor for the crystallographic planes (*hkl*) is given by equation (S1):

$$\left|F_{(hkl)}\right|^2 = F_{hkl}F_{hkl}^* = \left[\sum_{j=1}^{N} f_j \cos 2\pi(hx_j + ky_j + lz_j)\right]^2 + \left[\sum_{j=1}^{N} f_j \sin 2\pi(hx_j + ky_j + lz_j)\right]^2 \quad \text{(S1)}$$

where (*hkl*) represents the crystallographic planes, *N* represents the total number of atoms in a unit cell, f_j represents the atom scattering factor of the *j*-th atom in the corresponding unit cell. (*x, y, z*) represents the atom coordinate.

The structure factor *F* is generally given by a complex number. This factor represents the amplitude and phase of the scattered wave obtained from the summation of all atoms in the unit cell. The usefulness and application of equation (S1) can be fully appreciated by evaluating some actual cases [58]. For example, the structure factors of (001) and (002) for the face-centered (fcc) cell of HKUST-1 are given as follows:

$$|F_{HKUST-1(001)}|^2$$

$$= \left[\sum_{j=1}^{48} f_{Cu_j} cos\, 2\pi z_j + \sum_{m=1}^{192} f_{O_m} cos\, 2\pi z_m + \sum_{n=1}^{288} f_{C_n} cos\, 2\pi z_n\right]^2 + \left[\sum_{j=1}^{48} f_{Cu_j} sin\, 2\pi z_j + \sum_{m=1}^{192} f_{O_m} sin\, 2\pi z_m + \sum_{n=1}^{288} f_{C_n} sin\, 2\pi z_n\right]^2 = 0$$

$$|F_{HKUST-1(002)}|^2$$

$$= \left[\sum_{j=1}^{48} f_{Cu_j} cos\, 2\pi 2z_j + \sum_{m=1}^{192} f_{O_m} cos\, 2\pi 2z_m) + \sum_{n=1}^{288} f_{C_n} cos\, 2\pi 2z_n)\right]^2 + \left[\sum_{j=1}^{48} f_j\, sin\, 2\pi 2z_j + \sum_{m=1}^{192} f_{O_m} sin\, 2\pi 2z_m + \sum_{n=1}^{288} f_{C_n} sin\, 2\pi 2z_n\right]^2$$

$$= \left[-12.90 f_{Cu(002)} - 55.96 f_{O(002)} - 100.55 f_{C(002)}\right]^2 + [0]^2$$

where f_{Cuj}, f_{Oj} and f_{Cj} represent the atom scattering factor of the j-th Cu, O and C atom in the corresponding unit cell, and z represents the atom coordinates in the z-axis.

The results show that the reflections will be observed for planes of (002), but not the reflection for the (001) planes because its structure factor is equal to zero. In addition, the value of the sum of *cos* part in equation (S1) have relativity with the position of atoms in the lattice. And if the sum of the *sin* part in equation (S1) is equal to zero, this means the position of all atoms in the lattice must be satisfied as a top and bottom mirror set-out in the (001) plane.

As shown in Figure 3a (main text), for pristine HKUST-1, the structure factor of the (002) reflective plane is not zero, and PXRD shows a well-defined and sharp 002 reflection peak. However, the 002 peak has decreased to zero after Au(CO)Cl was loaded in the HKUST-1 SURMOF thin film (see Figure 3a, main text). This means that the structure factor of the (002) reflective plane needs to be zero. According to the equation (S1), we can calculate the structure factor of the (002) reflective plane of Au(CO)Cl@HKUST-1 with:

$$|F_{(002)AuClCO@HKUST-1}|^2 = \left[A_{(002)HKUST-1} + A'_{(002)Au(CO)Cl}\right]^2 + \left[B'_{(002)Au(CO)Cl}\right]^2 \cong 0 \qquad \text{(S2)}$$

where *A* represents the sum of *cos* part in equation (S1), *B* represents the sum of *sin* part in equation (S1).

The value of $B'_{(002)Au(CO)Cl}$ in equation (S2) needs to be equal to zero, as the (002) peak has dropped to near zero for the Au(CO)Cl loaded HKUST-1.

According to the experimental data, the 111 reflection is very weak in pristine HKUST-1 (see Figure 3b, main text). According to equation (S1), we further calculated the structure factor of the (111) reflective plane of HKUST-1 and Au(CO)Cl@HKUST-1 as follows:

$$\left|F_{(111)HKUST-1}\right|^2 = \left[A_{(111)HKUST-1}\right]^2 + \left[B_{(111)HKUST-1}\right]^2 \approx 0 \quad \text{(S3)}$$

$$\left|F_{(111)Au(CO)Cl@HKUST-1}\right|^2 = \left[A_{(111)HKUST-1} + A'_{(111)Au(CO)Cl}\right]^2 + \left[B_{(111)HKUST-1} + B'_{(111)Au(CO)Cl}\right]^2 \approx \left[A'_{(111)Au(CO)Cl}\right]^2 + \left[B'_{(111)Au(CO)Cl}\right]^2 \quad \text{(S4)}$$

Therefore, the main factor of the 111 reflection intensity is caused by the distribution and location/arrangement of Au atoms within the unit cell of HKUST-1. By using Bragg's law, wavelengths comparable to the atom spacing are scattered in a specular fashion by the atoms of a crystalline system, which undergo then constructive interference. Therefore, we can assume that the spacing of Au atoms or clusters is equal to the spacing of the (111) reflective planes (lattice planes) of HKUST-1. This lattice plane spacing d_{111} is 15.175 Å in the cubic Fm–3m HKUST-1 structure with *a* = 26.2832(2) Å from the cif-file with CCDC Refcode DOTSOV/CCDC no. 697917 [57] or 15.209 Å in the cubic Fm–3m HKUST-1 structure with *a* = 26.343(5) Å from the cif-file with CCDC Refcode FIQCEN [19] from the Cambridge Crystallographic Data Center, CCDC.

Infrared reflection absorption (IRRA) spectroscopy: all samples were recorded using an FTIR spectrometer (Bruker VERTEX 80) with a resolution of 2 cm^{-1} at an incidence angle of 80° relative to the surface normal. Liquid nitrogen is used to cool the mercury cadmium telluride (MCT) narrow band (4000–400 cm^{-1}) detector. Dry air was purged continuously through the spectrometer and sample compartment, which reduces the possibility of atmospheric water or CO_2 contamination on the samples. Samples were measured as long as the water absorption bands from ambient air disappeared (900–1300 scans). The data were processed using Bruker OPUS® software version 7.2. Perdeuterated hexadecane thiol SAM on Au/Ti/silicon substrates were used for reference measurements.

Scanning electron microscopy (SEM): High-resolution (HR-)SEM cross-sectional measurements have been performed on a Zeiss HR-SEM (Gemini Class) at 3-5 kV to check the continuity, compactness, and homogeneity of the different prepared (loaded and unloaded) HKUST-1 thin films.

Inductively coupled plasma optical emission spectrometry (ICP-OES): ICP-OES was performed using a Perkin-Elmer model Optima 8300 DV equipped with a GemTipTM Crossflow Nebulizer (Perkin Elmer, USA). All samples used in this work were dissolved in 65% nitric acid to give a sample concentration within the calibration limits.

S2 SEM image of Au(CO)Cl@HKUST-1 SURMOF (Figure S1)

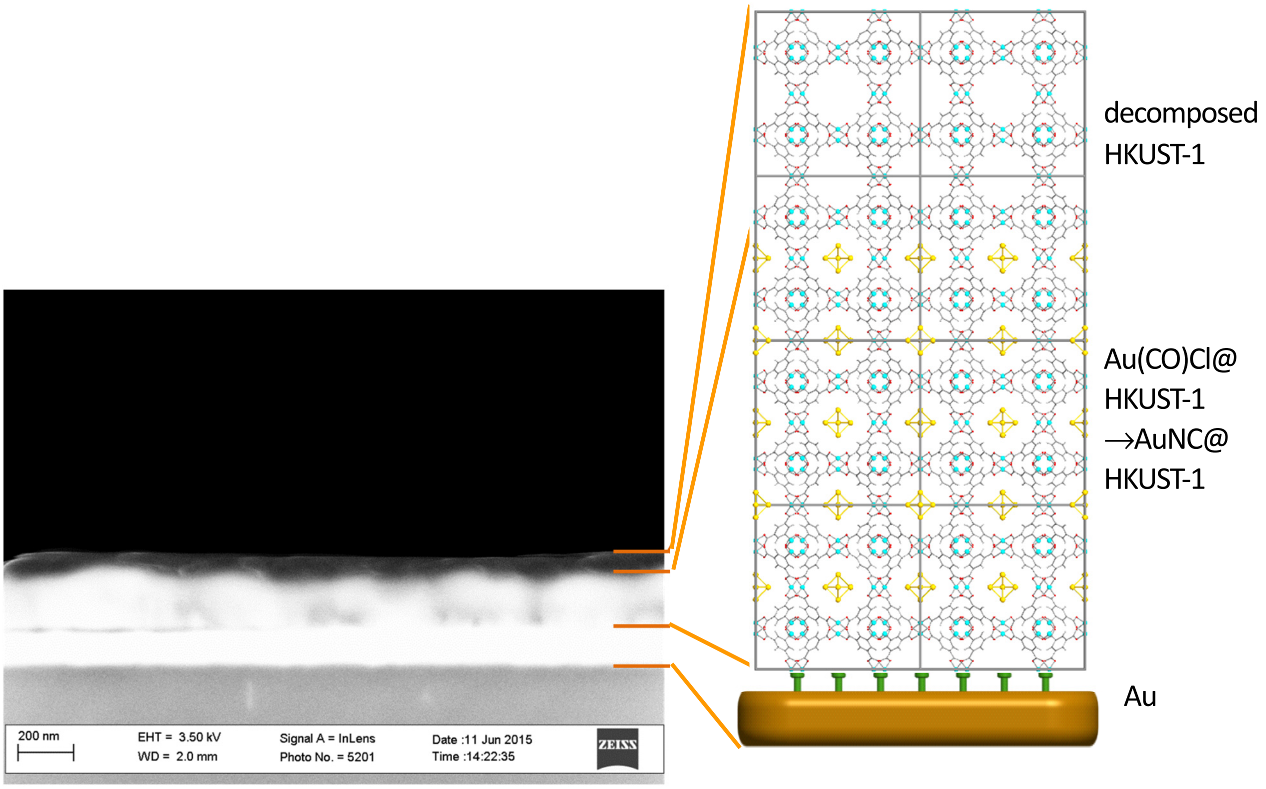

Figure S1. Left: HR-SEM image of ~45 layers Au(CO)Cl@HKUST-1 and ~20 layers of degraded HKUST-1. Right: Schematic depiction of these HKUST-1 layers filled with the gold clusters (derived from Au(CO)Cl) and the decomposed HKUST-1 layers. From experience with SEM images of SURMOFs the upper or outer layers are typically depicted as a "grey layer". Such a "grey layer" forms after a certain time upon interaction with ambient air. HKUST-1 is not moisture-stable [59,60].

Note that the image of octahedral Au clusters in the pores does not imply such Au_6 clusters in reality but is an artefact of the cubic space group symmetry, which positions an off-center Au atom by symmetry at the vertices of an octahedron.

S3 Gas-phase loading of Au(CO)Cl into HKUST-1 SURMOFs (Figure S2)

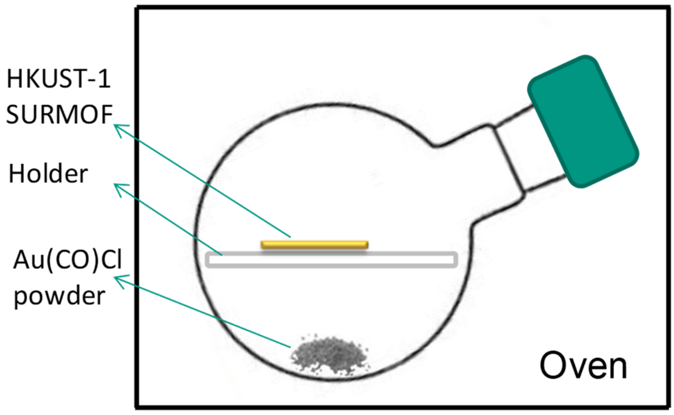

Figure S2. Synthesis scheme of gas-phase loading method.

S4 Powder X-ray diffraction (Figure S3 to Figure S6)

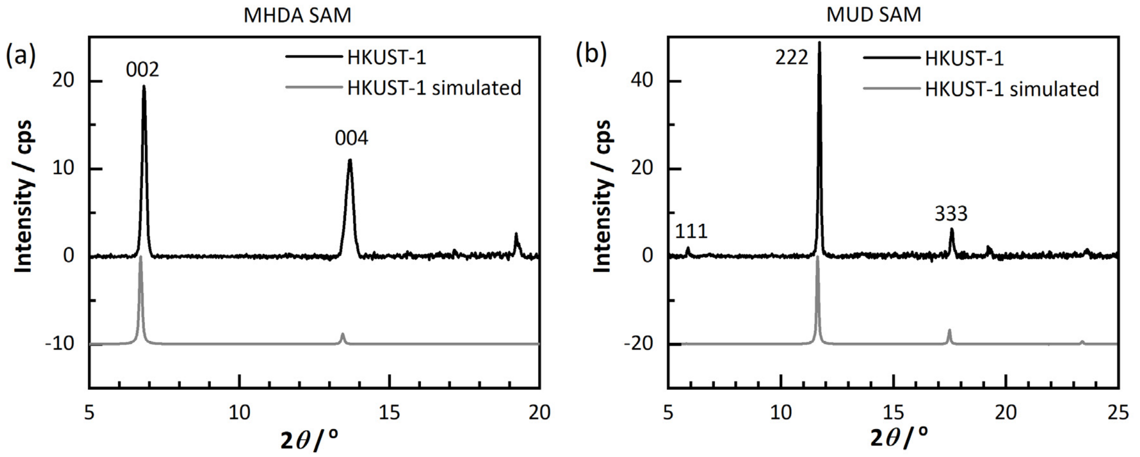

Figure S3. Powder X-ray diffraction (PXRD) patterns recorded for HKUST-1 SURMOF (**a**) grown on a MHDA SAM modified Au substrate; (**b**) grown on a MUD SAM modified Au substrate. Experimental diffractograms in black. Simulated diffractograms in gray. In (**a**) the simulation is for the preferred [001] orientation, in (**b**) the simulation is for the preferred [111] orientation. The 111 reflection in the simulation in (**b**) is present but of very low intensity. The simulated PXRD patterns have been calculated with the program MERCURY [56] using the cif file for HKUST-1 with Refcode FIQCEN [**Error! Bookmark not defined.**] from the Cambridge Crystallographic Data Center, CCDC. The crystal water molecule (O3) was removed from the cif file before the simulation, but the apical aqua ligands were kept. For the preferred orientation in MERCURY the March-Dollase parameter was set to 0.01. The reflections are designated by the Miller indices hkl.

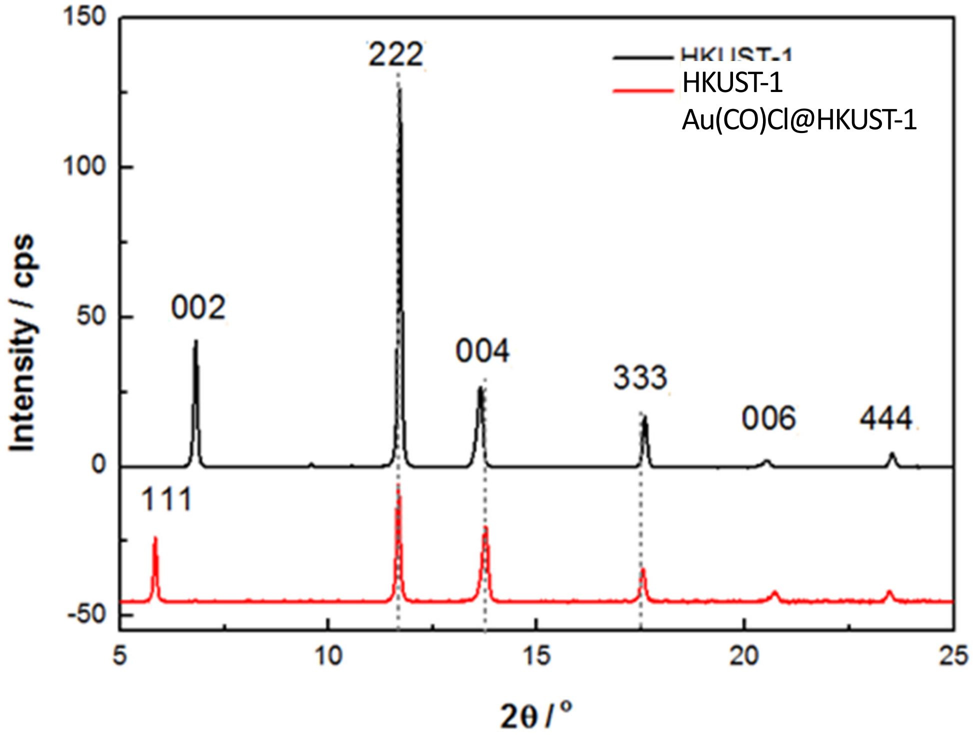

Figure S4. Powder X-ray diffraction (PXRD) patterns recorded for (a) empty (black) and after loading Au(CO)Cl (red) in the HKUST-1 SURMOF grown on a Si-wafer substrate. The reflections are designated by the Miller indices hkl.

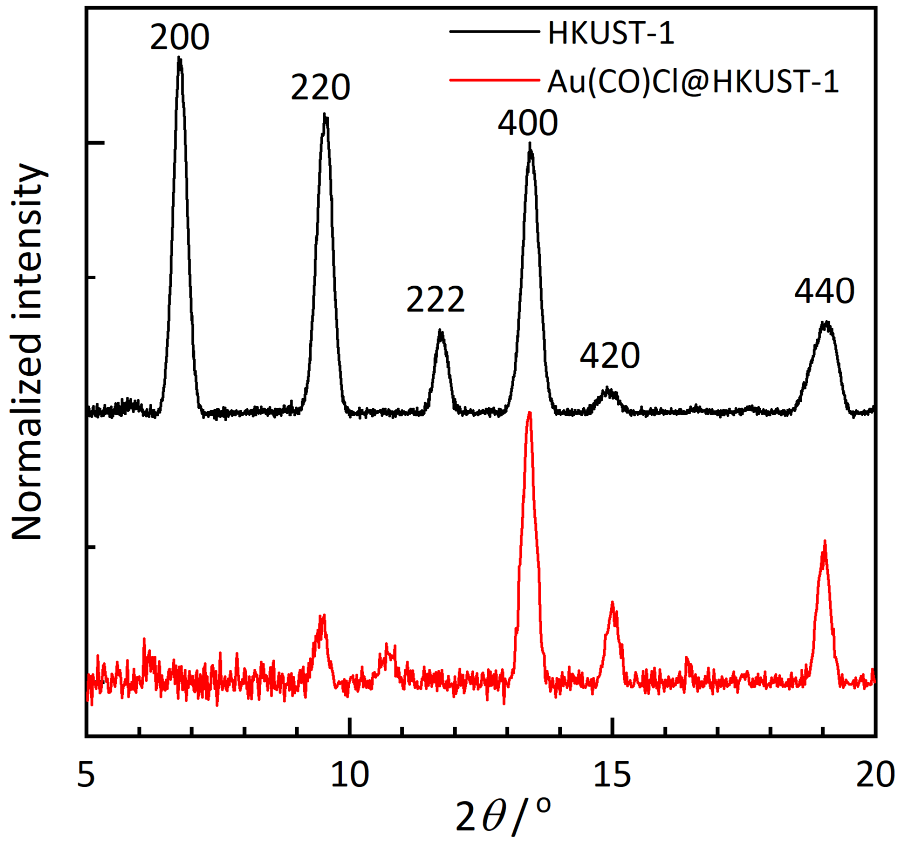

Figure S5. *In-plane* X-ray diffraction patterns recorded for empty HKUST-1 SURMOF (black) and after loading with Au(CO)Cl (red). The reflections are designated by the Miller indices hkl.

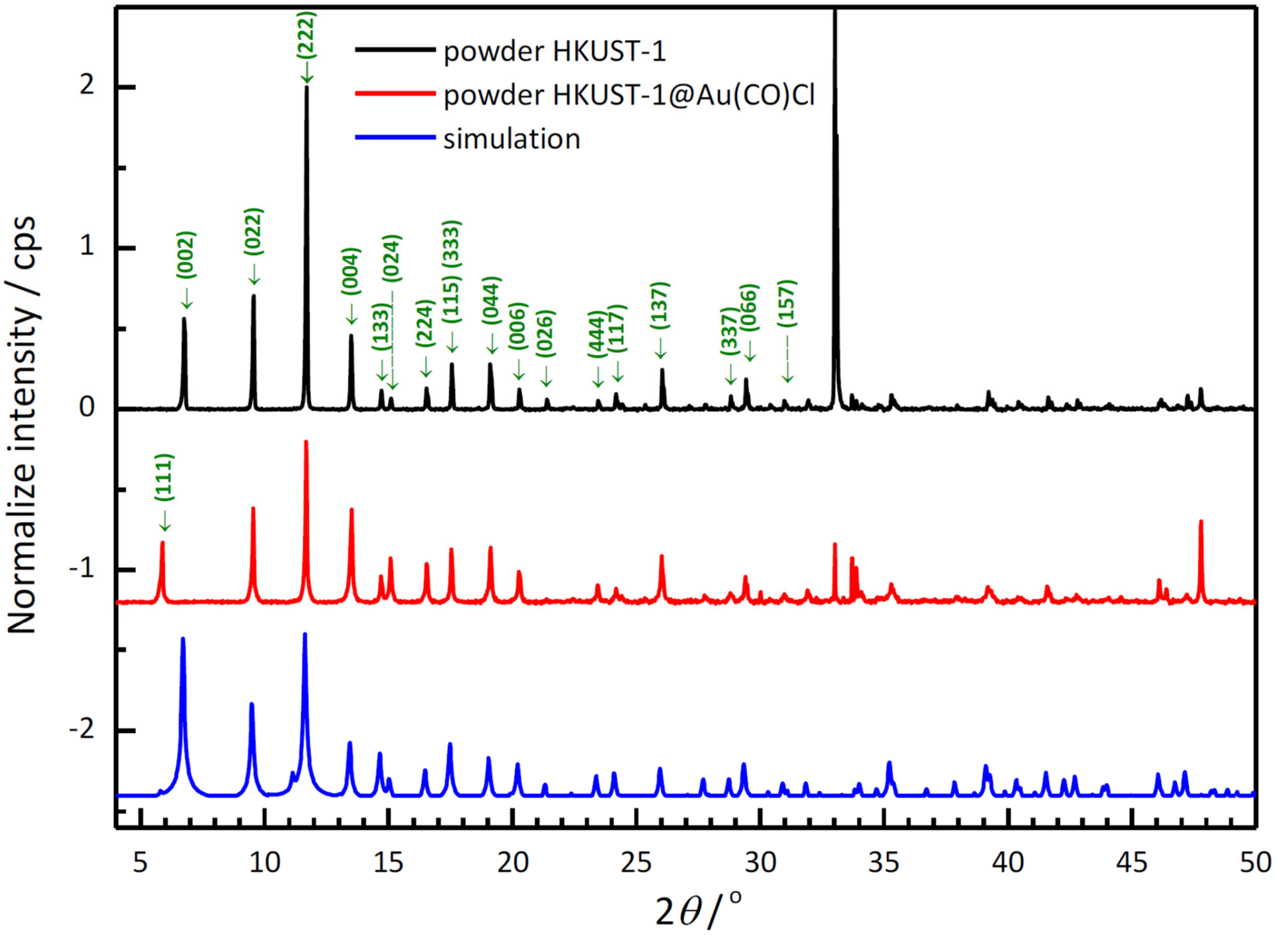

Figure S6. (**a**) Experimental powder X-ray diffractograms of empty (black) and Au(CO)Cl-loaded (red) HKUST-1 powder. (**b**) Calculated diffractogram (with λ = 1.5418 Å) of empty HKUST-1 using the cif-file with CCDC Refcode FIQCEN [**Error! Bookmark not defined.**] from the CCDC. (**c**) Calculated diffractogram for HKUST-1 (blue). The calculated blue diffractograms was plotted using a Gaussian profile function with 2theta steps of 0.02° and a frequency width at half maximum (FWHM) of 0.1°. The smaller experimental intensity than calculated for the (002) reflection below 2theta of ~7° can be explained from the flat sample holder and the Bragg-Brentano geometry for the measurement. At low angle this geometry broadens the X-ray beam spot on the sample such that only a fraction of the diffracted radiation reaches the detector giving lower than expected intensities. The reflections are designated by the Miller indices hkl.

S5 Time-of-flight secondary ion mass spectrometry and iso-surface (Figure S7, Figure S8, Table S1)

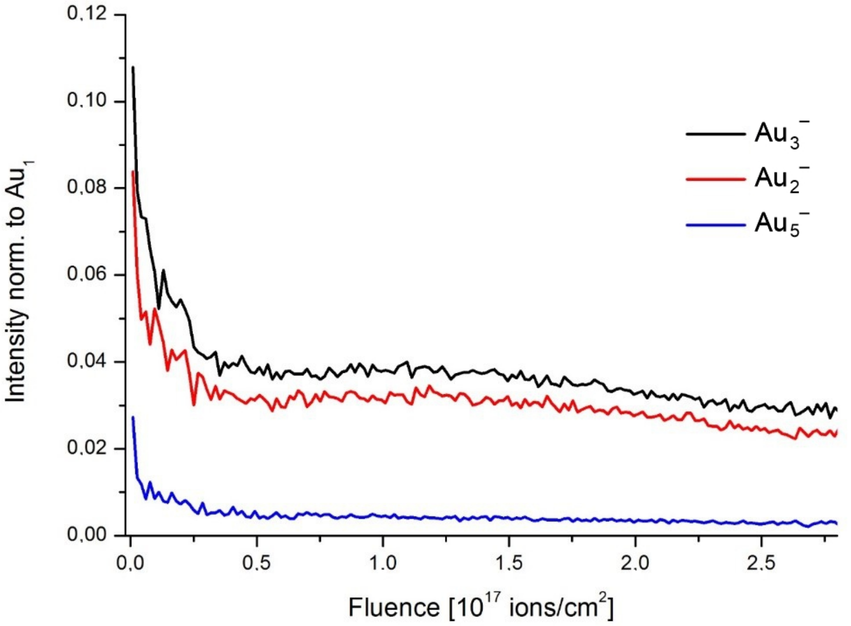

Figure S7. Relative signal intensities of Au_x gold clusters (x = 2, 3, 5) normalized to Au_1^- = 1 plotted against the Cs sputter ion fluence during depth profiling.

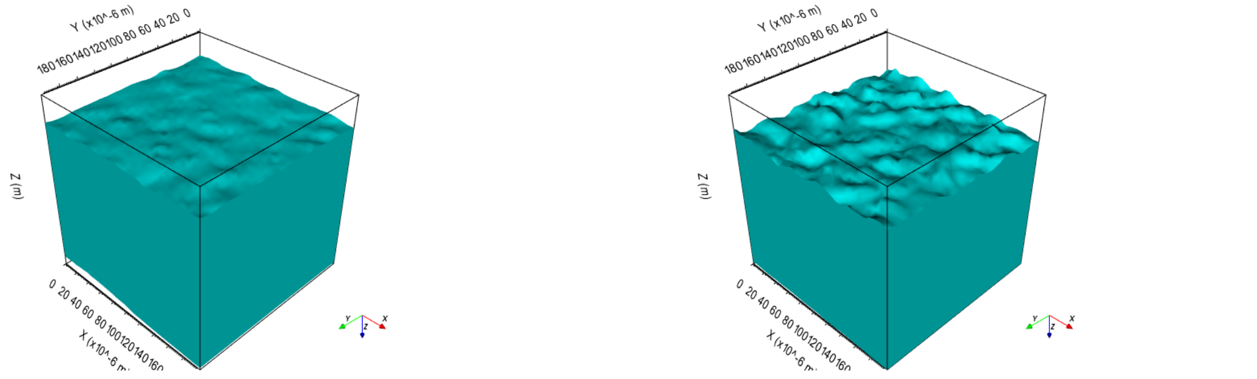

3D Render of Combined (^{65}Cu-, Cu-) 3D Render of Combined (Au5-, Au-, Au2-, Au3-, Au4-

(**a**) (**b**)

Figure S8. Iso-surface representation of the copper distribution (**a**) and Au_x distribution (**b**) in the analyzed SURMOF volume presented in Figure 4 in the main text. x = y = 200 µm, z-axis not to scale.

Table S1. Relative Au_x^- secondary ion intensities, normalized on Au_3^-, for metallic bulk gold and Au_x@HKUST-1 under 0.5 keV Cs dual beam erosion conditions.

	Au_1	Au_2	Au_3	Au_4	Au_5	Au_6	Au_7
Au@HKUST-1	26	0.84	1	0.085	0.11	0.004	0.004
Bulk Au	n.d. (detector saturation)	0.38	1	0.12	0.27	0.003	0.13

S6 ICP-OES and XPS (Figure S9 and Figure S10)

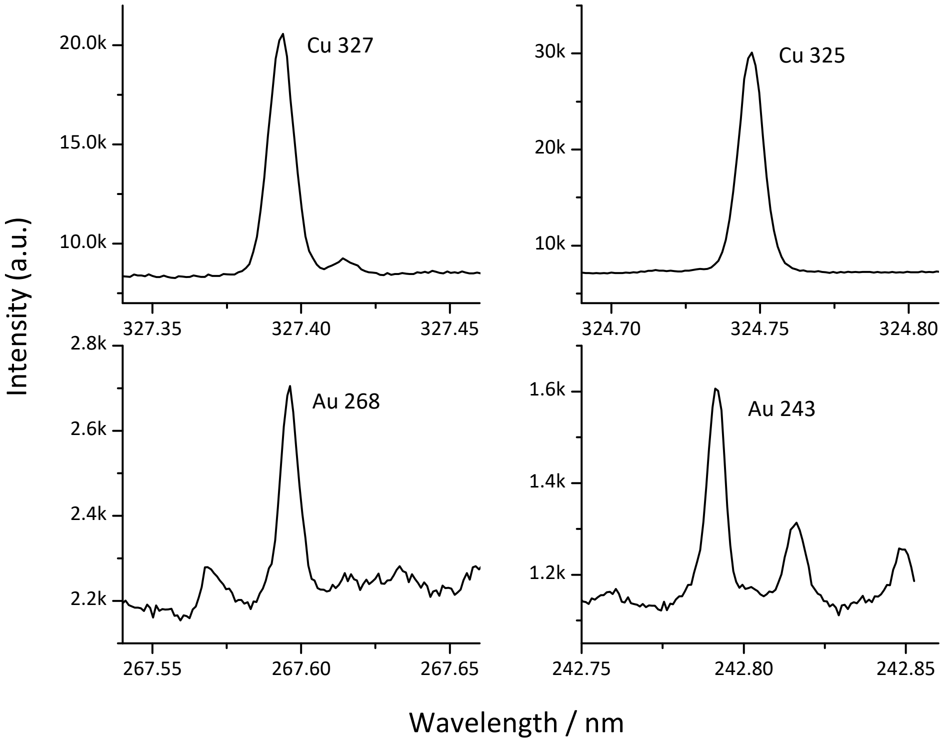

Figure S9. Inductively coupled plasma optical emission spectrometry (ICP-OES) of Cu (top row) and Au (bottom row) of the Au(CO)Cl loaded HKUST-1 powder sample. Shown are the UV emission bands with their wavelengths which were used for the quantitative analysis.

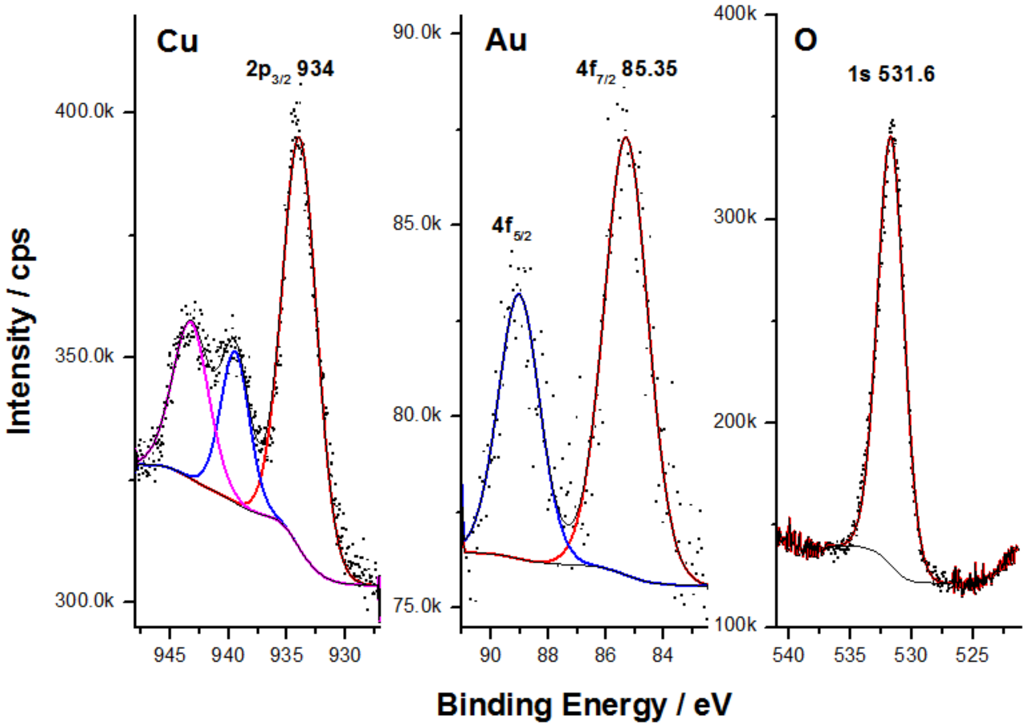

Figure S10. X-ray photoelectron spectroscopy (XPS) of Au(CO)Cl@HKUST-1 SURMOF thin film, grown on a Si wafer and measured directly after loading. The Cu $2p_{3/2}$ peak supports the assignment as Cu(II) for which 934 eV is a typical position [61]. In addition, Cu(II) has an observable collection of split satellite features up to 943eV, here at 938 and 934 eV. In Cu(I) compounds, there would be only a very weak satellite at 945eV [62]. The Au 4f region has well-separated spin-orbit components ($4f_{7/2}$ and $4f_{52}$, $\Delta \approx 3.7$eV) in an intensity ratio of about 1.3. For Au^0 the Au $4f_{7/2}$ photoelectron peak is located at a BE value between 83.9 and 84.4 eV. The Au $4f_{7/2}$ component at BE = 85.4 eV can be attributed to Au^{+1} species of Au(CO)Cl [63]. The O 1s peak at 531.6 eV confirms the presence of copper bound carboxyl oxygen, Cu–O_2C species [61,64].
